# A Comprehensive Review of Stimuli-Responsive Smart Polymer Materials—Recent Advances and Future Perspectives

**DOI:** 10.3390/ma17174255

**Published:** 2024-08-28

**Authors:** Alicja Balcerak-Woźniak, Monika Dzwonkowska-Zarzycka, Janina Kabatc-Borcz

**Affiliations:** Department of Organic Chemistry, Faculty of Chemical Technology and Engineering, Bydgoszcz University of Science and Technology, Seminaryjna 3, 85-326 Bydgoszcz, Poland; alicja.balcerak@pbs.edu.pl (A.B.-W.); monika.dzwonkowska-zarzycka@pbs.edu.pl (M.D.-Z.)

**Keywords:** stimuli-responsive materials, smart polymers, hydrogels, physical stimuli, chemical stimuli, biological stimuli, application of smart polymers

## Abstract

Today, smart materials are commonly used in various fields of science and technology, such as medicine, electronics, soft robotics, the chemical industry, the automotive field, and many others. Smart polymeric materials hold good promise for the future due to their endless possibilities. This group of advanced materials can be sensitive to changes or the presence of various chemical, physical, and biological stimuli, e.g., light, temperature, pH, magnetic/electric field, pressure, microorganisms, bacteria, viruses, toxic substances, and many others. This review concerns the newest achievements in the area of smart polymeric materials. The recent advances in the designing of stimuli-responsive polymers are described in this paper.

## 1. Introduction

The most common definition of polymers is that they are molecules consisting of repeating units characterized by various properties. This is a very general definition, however, that does not reflect the essence of polymers. The multitude of their types, methods of preparation, and application—topics on which paper can be written. Numerous studies in this area show that polymers offer more possibilities than limitations. They are an integral part of our lives, and it can undoubtedly be said that getting rid of them may cause a major regression in our civilization.

**Figure 1:**
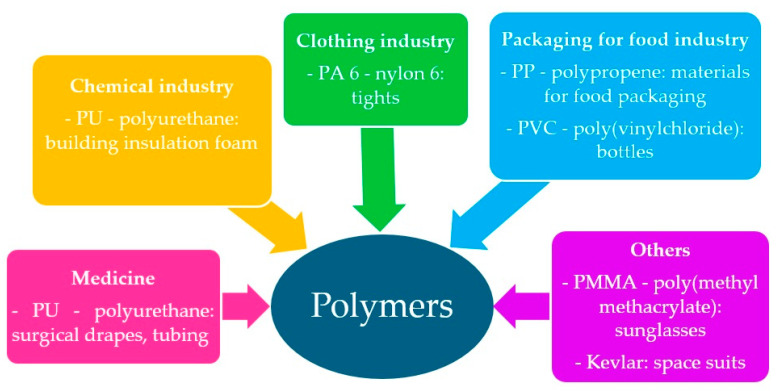
Potential use of polymers [1,2,3].

Being an element of our development, they are also subject to changes dictated by the environment, society’s requirements, and evolution. Some examples of polymer applications are shown in Figure 1 [1,2,3].

The presence of polymers in our lives is undeniable. Being part of our environment, they are also subject to some modifications. One result of this is the creation of new types of polymers and the emergence of a new group—smart (intelligent) polymeric materials [4].

The beginning of the era of intelligent materials dates back to 1950 when Katchalsky’s group started working on hydrogels [5]. Since then, interest in stimulus-responsive materials has been constantly growing. This fact is supported by numerous papers published every year.

**Figure 2:**
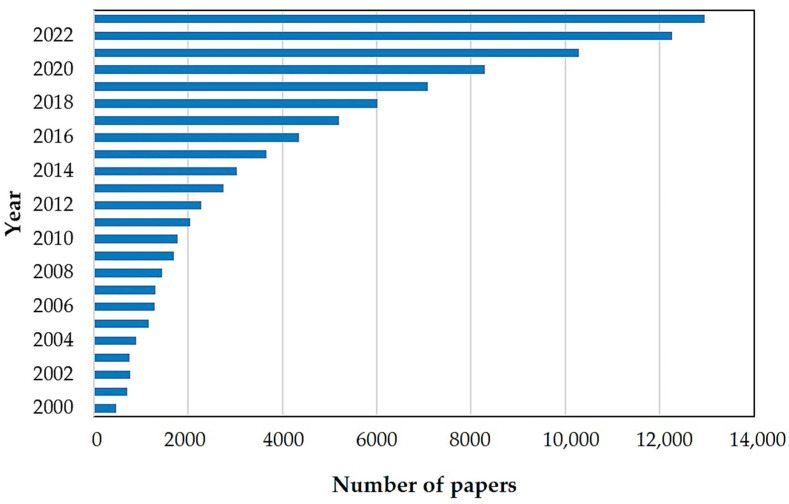
The number of publications published per year focused on stimulus-responsive materials, according to the Science Direct database. Keyword: smart material. Accessed on 8 July 2024.

As shown in Figure 2, in the years 2000–2011, a relatively small number of articles on the subject of smart materials were published (about 2000 articles). In the following years, this value gradually increased. After about 5 years, the number of papers had doubled. The year 2019 can be considered as a breakthrough in the research on novel stimuli-responsive materials. Significantly, from 2019 to 2023, about 6000 publications on this topic have been reported. The increased number of published works show how important searching for advanced materials for technology purposes is.

**Figure 3:**
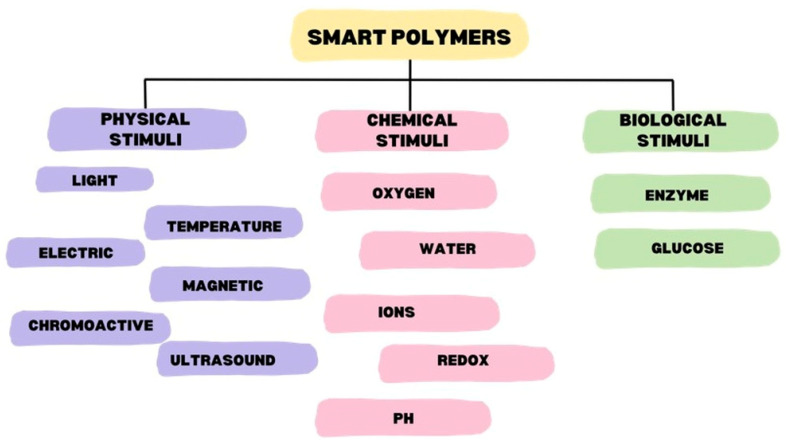
Classification of stimulants depending on the nature of the factor [5,7].

If we want to specify what these intelligent materials are, we can compare them to human intelligence or the recently increasingly mentioned artificial intelligence. In relation to this, intelligence means the ability to recognize, name, and respond appropriately to what is happening around us, solve a given problem, and learn. It is similar to smart polymers [4]. It defines a specific group of polymers that respond to external environmental factors by changing their physical or chemical parameters, which can be detected as changes in solubility, swelling, hydrophilicity/hydrophobicity, or micellization. This specific answer became the basis for designing materials useful in various industries. These factors may be physical, chemical, or biological in nature [5,6]. Sometimes, and more and more often, the term “multi-stimuli” can be heard, which means the ability of polymers to react to several factors [7]. Changes caused by a given factor are most often reversible, i.e., when they stop, the polymer begins to return to its original state [7]. A more detailed breakdown is shown in Figure 3 [5,7].

Smart polymers find a special area in medicine. Their specific features justifying this use are summarized below (Table 1) [8]. 

Taking into account the methods of synthesis of smart polymers, several basic ones can be distinguished:

▪ Traditional radical polymerization—conventional, which is characterized by mild reaction conditions and can be used in the presence of most monomers;
▪ Controlled radical polymerization—to which belong: (a) reversible addition-fragmentation chain transfer (RAFT) and (b) atom transfer radical polymerization (ATRP) [9].

On the other hand, due to their physicochemical form, stimuli-responsive polymers can also be classified into various groups, such as gels, solutions, self-organized clusters, coatings, solid materials, and others [5].

Another classification of intelligent polymers refers to the working mechanism of these materials. Taking into consideration this aspect, smart polymeric materials can be divided as follows: shape-memory polymers (SMP), self-healing materials, polymeric hydrogels, and other responsive polymers [4].

The future use of smart materials is primarily based on activities aimed at compliance with the principle of sustainable development. They are probably searching for new sources of natural polymers, modifying them, and looking for new intelligent properties. Although the nature of each material is different, their future seems bright due to the many advantages they present: reversible nature of changes and real-time response, they often respond to various environmental stimuli—expand their applicability to various fields by learning the response mechanisms—their reaction is clearly visible and predictable [10].

The aim of this review is to introduce the reader to the topic of smart polymers in a very short but clear way. The main idea of this paper is to characterize polymeric materials that show sensitivity to various stimuli. This article primarily describes the classification of smart polymers depending on the type of factor (physical, chemical, biological) to which they are responsive. Moreover, the application of these materials in various areas, as well as some examples of the latest achievements of various types of smart materials, such as hydrogels, shape-memory polymers, self-healing materials, and others, are presented.

This review does not focus on a specific type of smart polymer but describes the topic comprehensively. In order to highlight the huge potential of this group of smart materials, their selected advanced applications in medicine, chemical industry, agriculture, and modern technologies are presented.

## 2. Physical Stimuli

### 2.1. Light-Responsive Polymers

Multi-advantage, light-responsive polymers are characterized by biocompatibility, a high degree of solubility in water, biodegradability, and the ability to spatial and temporal control in response to light of a specific wavelength, intensity, and area of exposure [11]. The disadvantages include the possibility of leaching chromophoric substances from the hydrogel as a result of swelling [8]. Methods for the synthesis of light-responsive polymers assume the use of two mechanisms:

▪ photocleavage—which involves the occurrence of chemical changes creating a physicochemically changed product;
▪ photochromic—based on the occurrence of isomeric changes based on *cis*-*trans* isomerism, intramolecular transfer of groups or a hydrogen atom, or pericyclic changes [11].

The great interest in this type of polymer results from the advantages presented by the stimulating factor—light. It is cheap, safe, and readily available [12]. A feature necessary to produce light-responsive polymers is the presence of compounds that respond to a specific wavelength of light. Among the most common are azobenzenes, stilbene, cyanostilbene, stiff-stilbene, diarylethene, spiropyrans, hydrazones, coumarins, and others [13]. Depending on the chromophore used, a different wavelength range is preferred. For medical purposes, UV radiation is avoided, and systems that respond to visible light are necessary [8]. The response of chromophore groups may be based on one of three presented mechanisms: photolysis, photoisomerization, and photorearrangement [12]. Recently, much attention has been paid to derivatives of *o*-nitrobenzyl alcohol as a presented group photorearrangement. It is one of the best-tested compounds and is used in many industries, which responds to radiation in the range of 300–365 nm [14].

Polymeric materials can show sensitivity to NIR radiation emitted by the laser. This property of stimuli-responsive materials has also gained importance in medicine, especially in photothermal therapy (PTT) [15]. In the literature, many examples of the use of smart hydrogels in this field were described. The generated thermal effect damages infected cells. What is important, NIR light emitted by laser exerts not only a photothermal effect but also can stimulate the systems for drug release [16]. For example, Fu and colleagues [17] developed a thermos-sensitive, hydrogel-enabled thermostatic PTT system for effective healing of wounds, which are infected by bacteria. On the other hand, Algi and others [18] proposed poly(2-hydroxyethyl methacrylate) hydrogels combined with squaraine dye for photothermal/photodynamic therapy and as a drug delivery system. The synthesized hydrogel induced hyperthermia upon laser irradiation with 808 nm and generated ROS.

### 2.2. Temperature-Responsive Polymers

As the name suggests, temperature-responsive polymers are able to change their properties (often solubility) in response to changes in temperature (heating or cooling) in the external environment. Temperature is a parameter that can be measured and monitored very easily, and the systems for doing so are well understood. For this reason, it is often used in the production of smart polymers [11]. A given material can acquire or change its temperature-responsive properties by adding additional substances to the system, such as plasticizers, salts, and surfactants [19]. The reaction is based on the transition from the sol-gel state. A distinguishing feature of these polymers is the presence of lipophilic groups: methyl, ethyl, or propyl. An example of a temperature-responsive polymer is poly(*N*-isopropylacrylamide)—PNIPAAm, poloxamers, and prolastin [9].

Parameters associated with the assessment of this type of polymer are:

▪ UCST—upper critical solution temperature. UCST—is poorly known.
▪ LCST—lower critical solution temperature—indicates the maximum temperature at which the polymer is soluble, and one phase can be observed. Above it, phase separation takes place [9]. LCST-polymers are well known. The existence of a single phase comes from the interactions between the polymer and solvent units. The most common are hydrogen bonds with water [9,11,20].

**Figure 4:**
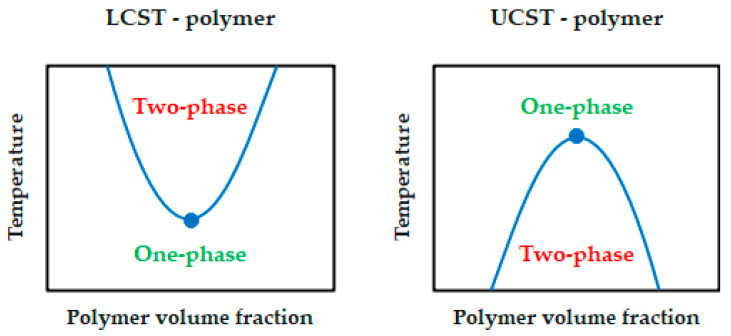
Schematic diagram showing the difference between LCST and UCST polymers [20].

In other words, heating causes phase separation in LCST polymers and single-phase formation in UCST polymers. The differences between LCST and UCST polymers are better shown with an example diagram (Figure 4).

**Figure 5:**
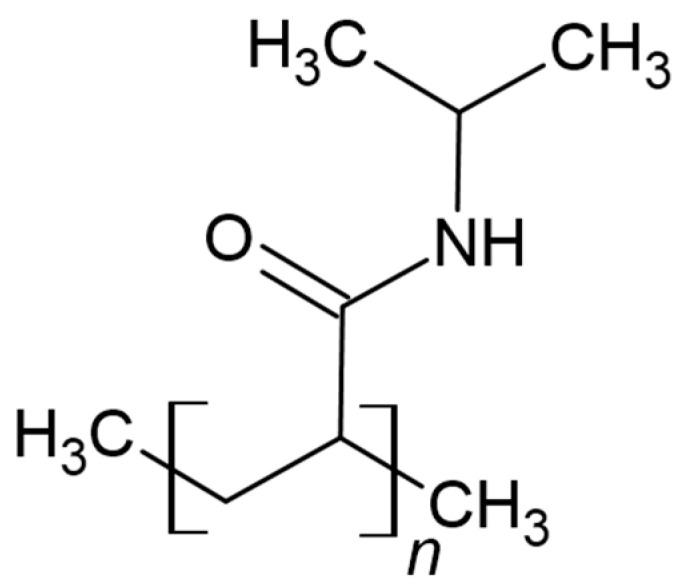
Chemical structure of PNIPAAm [21].

As far as the reaction mechanism for the development of thermo-responsive properties of polymers is concerned, the formation of bonds between molecules of hydrophilic/hydrophobic groups and water molecules is considered appropriate. Of course, the formation of bonds and the occurrence of changes can be observed in the form of changes in weight, color, and transparency [14]. The first polymer used in the design of thermo-responsive materials was PNIPAAm (poly(*N*-isopropylacrylamide)). The chemical structure of this compound is shown in Figure 5 [21].

Its use is due to the fact that it has a similar LCST temperature—32–33 °C—close to the human body temperature.

### 2.3. Electric Field-Responsive Polymers

Electric field-sensitive polymers are characterized by changes in physical properties in response to small changes in electric current. The content of a large number of easily ionizable groups makes them sensitive to pH changes. Changes in the electric field have been due to the conversion of electrical energy into mechanical energy. Considering one of the possible applications of electric-field-sensitive polymers—drug delivery—the reaction mechanism involves breaking hydrogen bonds in the existing structure under the influence of an electric current and releasing the drug at the target site [8,9]. Other uses of electroresponsive polymers are robotics, electrochromic devices, actuators, energy harvesting, or electroconductive scaffolds with use in tissue engineering [22,23]. Their main division assumes the existence of two groups of materials:

▪ Ionic EAPs (electro-active polymers)—the electric field causes a change in local ion concentrations and the occurrence of electroreactivity. Their characteristic feature is low reaction speed, low reactivity, and the need to use low voltages;
▪ Dielectric EAPs—where the response arises as a result of electrostatic forces arising between two electrodes applied to the system. Their characteristic feature is high reaction speed, high reactivity, and the need to use high voltages [23].

Among the polymers used to produce electro-responsive polymers, the following are of particular importance:

▪ Polypyrrole (PPy)—characterized by high biocompatibility and high conductivity;
▪ Polyaniline (PANI)—characterized by high chemical stability, good processability and conductivity;
▪ Poly(3,4-ethylene dioxythiophene) (PEDOT)—which, in addition to being biocompatible and highly conductivity, is also hydrophobicity;
▪ Chitosan (CS)—is a natural—sourced polymer characterized by high availability, biocompatibility, microbiological activity, the ability to form a gel, and ease of processing [8,22].

### 2.4. Magnetic Field-Responsive Polymers

Magnetic field-responsive polymers are a group of materials that have the ability to change their parameters, such as density, optical properties, and shape, in response to a magnetic field [24]. Obtaining these specific properties can be achieved by adding magnetic particles (which are activated with a magnetic field), for example, magnetite—Fe_3_O_4_, maghemite—γ-Fe_2_O_3_, to the formulation combined with polymers such as poly(ethylene glycol)—PEG, dextran, poly(vinyl alcohol)—PVA, poly(ethylene imine)—PEI [19,25]. The particles added to the formulation may be referred to as soft (low coercivity, a change in material properties occurs after the application of a magnetic field as a result of dipole-dipole interactions and the generation of internal heat) or hard magnetic particles (deformations can occur as a result of internal interactions between molecules even in the absence of a magnetic field) [26]. The advantage of magnetoresponsive polymers is the possibility of spatiotemporal control, while the main disadvantage is the possibility of aggregation of materials [25].

One of the areas of interest in these materials is medicine and drug delivery. Thanks to the use of a magnetic field, it is possible to improve the efficiency of drug release, which significantly improves the effects of treatment [24]. Their versatile use (thanks to the use of a magnetic field and not the sometimes destructive effect of heat or noise)—(soft robotics, shape memory polymers, biomedicine) is due to the advantages they present: ease of use, rate of activation and response, compatibility with the environment [4,26]. An example of a polymer that responds to a magnetic field is PNIPAAm hydrogels containing the ferromagnetic material PNIPAAm-co-acrylamide [8].

### 2.5. Chromoactive Polymers

This group of materials is able to change its color under the influence of external factors. These factors can be classified into three groups:

▪ Photochromic materials—which are distinguished by a reversible color change when exposed to light with a high UV content;
▪ Thermochromic materials—the color change occurs as a result of temperature. The dye used determines the permanent or transient occurrence of the color;
▪ Electroactive materials—the occurrence of a potential difference triggers a color change and absorption spectrum [27].

### 2.6. Ultrasound-Responsive Polymers

The term ultrasound should be understood as a wave caused by alternating current resulting from the mechanical vibration of a piezoelectric material. Depending on the frequency, waves can be distinguished: low (<1 MHz), medium (1–5 MHz), high (5–10 MHz). Ultrasounds can affect the material in the following ways: (a) thermal—when an increase in temperature is observed; (b) nonthermal, also known as cavitation—during this process, ultrasonic vibrations cause gas bubbles to form [23]. 

The use of these types of polymers may be particularly useful, and sometimes be the only option when other stimulants fail. This may mean biomedical use, where, for example, sometimes cooling or heating is not advisable. In this case, ultrasound may be used effectively to release the drug. The main advantage of systems using ultrasound is that there is no need to add foreign ingredients, so the polymer becomes more environmentally friendly. Other advantages include it can be used in opaque materials, can cover small areas, and can be controlled spatiotemporally, economically, and highly accessible [25,28]. The forms of polymers capable of ultrasound response are gels, solids, micelles, and coated microbubbles. In the case of micelles, the use of micelles causes disintegration, releasing a specific factor [29]. An example of an ultrasonic-responsive polymer is dodecyl isocyanate-modified PEG-grafted poly(2-hydroxyethyl methacrylate), polyglycolides, or polylactides [8,29]. The use of ultrasound produces specific responses, including streaming, cavitation, structural vibrations, radiation force, and scattering [28].

## 3. Chemical Stimuli

Chemical stimuli can also modify the properties of polymeric materials. Chemical factors induce changes in the interactions between polymer chains or between polymer chains and solvent molecules. Depending on the type of chemical stimulus, conformational changes, shrinking or swelling of the polymer material, and others may occur [5,30].

### 3.1. pH-Responsive Polymers

Materials of this type have aroused considerable interest among many groups of researchers due to the possibility of versatile application [19]. A characteristic and distinguishing feature of pH-responsive polymers is the presence of one of the groups: acidic or basic, whose task is to take or donate protons in response to a change in pH. The overall change in charge changes the structure of the polymer chain which manifests itself in changes in properties (solubility), configuration, or surface activity [8,19]. The reaction mechanism is as follows:

(a) In the case of acidic polymers—protons attach at low pH and release of protons at high pH;
(b) Basic polymers react by ionization/deionization in the pH range of 7–11 [19].

Polymers that show pH sensitivity can be classified into the following categories:

(1) Natural origin polymers:
  ▪ Alginates: acidic polysaccharides with pKa ca. 3–4 (resulting from the presence of -COOH groups). In the presence of divalent cations (Ca^2+^, Ba^2+^, Sr^2+^ and Zn^2+^) it gels gently;
  ▪ Hyaluronic acid—a linear polysaccharide that has a pH of 3–4. It absorbs water up to 1000 times its volume, creating a loose network;
  ▪ Chitosan—a polysaccharide that owes its pH sensitivity to the presence of amino groups in its structure. At low pH conditions, the amino groups are protonated, which triggers the ability to dissolve at low pH, and poor solubility at high pH;
(2) Synthetic polymers—which include two types of compounds, such as:

(a) Polymers containing a pendant group:
  ▪ Polyacids—which contain acidic groups in their structure, e.g., carboxylic: poly(acrylic acid)—PAAc, boronic: poly(vinylphenyl boronic acid)—PVPBA, phosphoric: poly(ethylene glycol acrylate phosphate)—PEGAP and sulfonic acid: poly(vinyl sulfonic acid)—PVSA;
  ▪ Polybases—which contain the following groups in their structure, e.g., amino: poly[(2-dimethylamino)ethylmethacrylate]—PDMA, pyridine: poly(4-vinylpyridine)—P4VP, imidazole group: poly(*N*-vinylimidazole)—PVI.
(b) Polymers containing labile acid/base linkage—This group contains polymers that are capable of breaking bonds under the influence of pH change, for example:
  ▪ Hydrazone (decomposing at pH 5.5);
  ▪ Imine (decomposing at pH 5);
  ▪ *Cis*-aconityl (decomposing at pH 4) [31].

There is a great interest in pH-responsive materials in the medical industry. Cancer cells have an acidic pH, which is the basis for triggering a response in the material [25]. This relationship is known as the Warburg effect. The rapid proliferation of cancer cells disrupts the supply of blood and oxygen. Cells produce energy through glycolysis by producing lactic acid, which lowers the pH [21].

### 3.2. Ion-Responsive Polymers

This is a group of materials that respond to changes in ionicity in the surrounding environment. They exhibit reversible physical and chemical reactions to fluctuations in pH or the number of ions. A change in the ionic strength in the surrounding environment changes the interactions between the ions in the solution and the ions in the polymer, leading to swelling/dehydration. Examples of polymers that respond to ions are alginate (Ca^2+^) and chitosan (Mg^2+^) [8].

### 3.3. Redox-Responsive Polymers

The term redox-responsive polymers can be used to describe materials in a fairly broad group. They respond with specific reactions to changes in the redox state. The reaction occurs as a result of the presence of oxidants or reducers in the environment. These changes can be caused by many factors, including temperature, pH, and light [5]. The scope of application of these materials is wide, but the topic of hydrogels and the release of drugs as a result of redox-responsiveness is often encountered. The Kilic–Boz group addressed the issue of the release of biomolecules (bovine serum albumin) from hydrogels as a result of the action of thiol-containing agents (dithiothreitol—DTT and L-glutathione—GSH). The redox response was possible due to the introduction of disulfide and organometallics to materials [32,33]. In the case of gels, the response may include changes in color, chiral structure, phase, and fluorescence [34]. An example of a polymer reacting to redox reaction is PNIPAAm hydrogel containing *tris*(2,2-bipyridyl) ruthenium (II) (Ru^2+^ → Ru^3+^) [8].

### 3.4. Water-Responsive Polymers

Water-responsive (WR) polymers are materials that respond to changes in humidity or the amount of water in the environment. Other names often associated with these polymers are humidity-responsive or humidity/water-responsive). These changes can be noticed as swelling or shrinking of the material [28]. Generally speaking, they are able to generate mechanical energy using the available chemical potential of water [19]. A polymer with a porous structure that allows it to react to moisture. It allows water to penetrate between the fibers [4]. In the development of water-responsive polymers, polymers with a hierarchical structure play an important role in their production, ensuring appropriate mechanical and physiological properties of the structure. The main example of a substrate is cellulose.

Among the fabrication methods of WR materials, there are several basic ones:

▪ Solvent-casting—which involves creating a solution with active ingredients (active layers—most often chitosan or sodium alginate), pouring it onto a film (passive layers—most often poly(vinyl chloride) or poly(propylene)) and dry;
▪ Spin coating—an example of which is the formation of a water-responsive poly(3,4-ethylenedioxythiophene):polystyrene sulfonate/poly(dimethylsiloxane)—PEDOT:PSS/PDMS actuator;
▪ Photolithography;
▪ 3D printing;
▪ Fibre spinning [28].

Due to the specificity of the mechanism, special attention is paid to hydrogels. The most common components are polymers: PEG—poly(ethylene glycol), HEMA—2-hydroxyethyl methacrylate, pAAM—poly(acrylamide) and hyaluronic acid gels [33].

### 3.5. Reactive Oxygen Species-Responsive Polymers

Oxygen is a factor produced in every living cell as a result of intracellular respiration by mitochondria. However, small amounts of it do not have negative effects. However, increasing the concentration may contribute to homeostatic disturbances, which may negatively affect lipids or DNA. This reaction became the basis for the production of oxygen-reactive, species-responsive polymers, particularly useful in medicine [35]. Forms of ROS-responsive polymers include nanoparticles, hydrogels, and scaffolds. Their reaction involves degradation as a result of ROS or changes in solubility. Among the polymers presenting the oxidation response, there are polymers containing the following groups:

▪ Thioether group, e.g., poly(propylene sulfide)—PPS;
▪ Selenium;
▪ Tellurium;
▪ Poly(thioketal);
▪ Phenylboronic acid/phenylboronic ester.

## 4. Biological Stimuli-Responsive Polymer Materials

The properties of polymer materials may undergo functional changes under the influence of biological factors. Among the biological factors that can influence the properties of polymers are glucose and enzymes.

### 4.1. Glucose-Responsive Polymers

The development of glucose-responsive polymers offers hope for potential use in medicine in the treatment of people with diabetes problems. Their work is to respond to glucose levels and control the release of insulin. The main disadvantage is the short response time.

### 4.2. Enzyme-Responsive Polymers

Enzyme-responsive polymers are a class of materials that can react to the presence of enzymes in a specific way. Taking advantage of enzyme features such as the ability to catalyze physicochemical changes and biorecognition, they are very useful in designing systems that are particularly useful in places where there is overexpression of a given enzyme. The advantages of enzyme-responsive polymers include specificity of action in the cell, the possibility of spatiotemporal control of secretion, a large number of enzymes that can be used in a variety of applications, increased permeability, and increased resistance to degradation. The main disadvantages include the possibility of premature release of the charge—when an enzyme with a similar structure is present and the possibility of releasing the charge before reaching the target [36].

## 5. Multistimuli Polymer Materials

As mentioned above, smart polymers can be divided into two main groups: single- and multiresponsive. Multiresponsive polymer materials show sensitivity to a combination of two or more stimuli, e.g., light, mechanical force, temperature, pressure electric/magnetic field, pH, concentration gradients, humidity, biological environment, and many others. Significantly, physical, chemical, and biological factors may cause permanent or reversible changes in polymeric properties [36]. For example, materials that respond to a combination of more than one factor, such as light/temperature, enzyme/pH, pH/temperature, temperature/pH/redox, and many others, have been described in the literature [21,37].

The design of multistimuli polymers can be very complex and demanding. The development of smart materials often requires multidisciplinary approaches, including knowledge of many areas. However, the fabrication of functional polymers involves the introduction of innovations and influences the development of advanced technologies. The incorporation of suitable groups into the polymer chain affects the response of materials to multistimuli [38,39].

Multisensitive polymers show significantly greater functionality and capability than single stimuli materials. Each stimulus may cause a reaction individually or cause interdependent changes. Interdependent stimuli can induce reactions occurring successively, or the first reaction might enhance or alter the properties of polymer materials [39,40,41]. Polymeric materials that are sensitive to many stimuli are very popular due to their multidirectional application capabilities in various fields of science and technology [8,42,43,44,45].

## 6. Application of Smart Polymer Materials—Latest Advances

**Figure 6:**
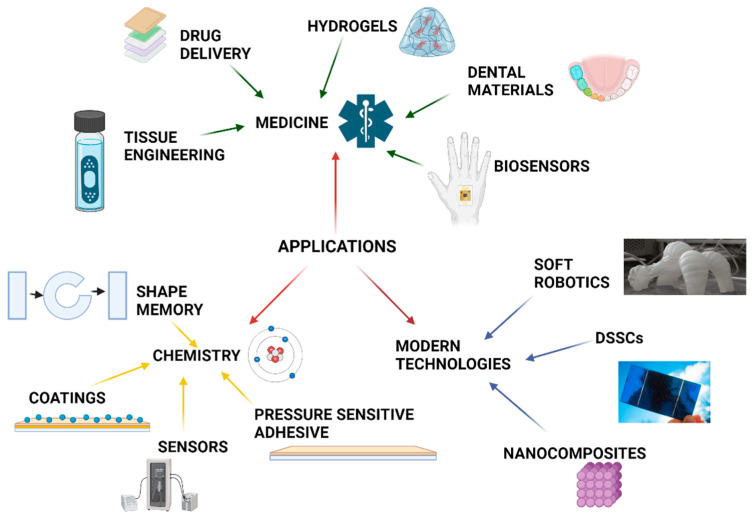
Application of smart polymer materials in various areas.

Smart polymers are used in many areas, such as medicine, the chemical industry, and modern technologies. Taking into account the ability to react to material for stimuli, the designed polymer can be applied in a dedicated area. The application fields of smart polymeric materials are shown in Figure 6.

Figure 6 shows that the areas of application of stimuli-responsive polymers are almost unlimited. Moreover, the intensive research toward novel stimuli-responsive materials gives a chance to expand this group of smart materials. 

### 6.1. Medicine

One of the main applications of smart polymers is medicine. The increased demand for specialized polymeric materials that could cure various diseases and thus significantly improve the quality of life has led to the rapid development of functional biomaterials. Polymeric materials showing sensitivity to various stimuli are used in drug delivery systems [8,46,47], tissue engineering [48,49,50], bioimaging [51,52,53], gene carriers [54], cell culture [55,56,57], and in the production of various types of medical products or devices [58,59]. Generally, there are a wide group of smart polymers that are used in the medical sector, e.g., hydrogel dressings [60,61], implants [62,63], tissue adhesives [64,65], ocular lenses [66,67], biosensors [68,69], and others. 

It should be pointed out that the most commonly used type of smart polymer in the medical field is in the form of hydrogels. Hydrogels are a three-dimensional polymeric network with a high capacity to absorb a huge amount of water or other aqueous solution. Generally, this class of polymers is considered one of the most promising materials for medicine. This is due to their specific physicochemical, biological, and mechanical properties, such as hydrophilicity, biocompatibility, viscoelasticity, softness, biodegradability, and many others. In other words, hydrogels show a great similarity to human tissues, which makes them ideal candidates for healthcare applications [4,70,71,72]. 

Taking into account various factors (e.g., source of origin, configuration of chains, sensitivity to stimuli, etc.), hydrogels can be divided into various groups [73]. An important impact on the final properties of hydrogel materials is the method of their synthesis. There are two basic routes of obtaining hydrogels, i.e., chemical and physical crosslinking [74]. Physically, crosslinks include intermolecular reversible interactions, such as electrostatic/ionic interactions, hydrogen bonding, hydrophilic/hydrophobic interactions, as well as crystallization, stereo complex formation, metal coordination, polymerized entanglements, etc. The most important advantage of this type of crosslinking is the biomedical safety of created materials owing to the absence of chemical crosslinkers. Moreover, the hydrated polymers can show responsivity to various stimuli and self-healing abilities [74,75,76].

Chemical crosslinking comprises photopolymerization, “click” chemistry (Michael type addition, Diels-Alder reaction), oxime formation, Schiff base linkage, and enzyme-induced reaction [74]. Compared to the physical crosslinks, chemically crosslinked hydrogels are formed by strong and permanent covalent bonds among polymeric chains and create a stable network. This structure affects increased mechanical resistance. Moreover, these hydrogel materials show enhanced stability under physiological conditions and tunable degradation behavior. It should also be pointed out that the type and degree of crosslinking influence many properties of hydrogels, e.g., swelling ability, elasticity, and others [74,77].

Polymers fabricated for medical purposes must strictly meet specific requirements, such as biocompatibility, nontoxicity, nonmutagenicity, etc. [78,79,80]. This is due to the fact that these materials must very often act as appropriate analogues of soft tissues, organ fragments or bones. For this reason, designing new polymer materials for medical applications requires appropriate matching of physicochemical and biological properties to their functionality and application [81,82].

Numerous papers on the use of smart polymers in drug delivery systems emphasize the importance of this topic. These works present an innovative approach to a method of precise drug (s) delivery to target cells [83]. Drug delivery systems (DDSs) are a pharmaceutical product (formulations or devices) that enables the introduction of targeted therapeutics, which results in improved safety and efficiency of used substances. What is important is that drug supply via DDSs is controlled in terms of rate, time, and concentration of medicament [84,85,86]. Therefore, this way of drug delivery shows a significant advantage over conventional forms, such as solutions, pills, and semi-solid preparations. Apart from the possibility of multidimensional drug release control, the advanced DDSs protect the drug from unfavorable changes in the biological environment, increasing their efficiency, as well as minimizing the side effects [87].

Smart polymers, especially sustained-release drug delivery systems based on hydrogels, play an important role in cancer treatment [88]. Encapsulating the anticancer drug within the hydrogel network can protect it from rapid degradation, immune rejection, and other unfavorable factors. This hydrogel capsule provides not only a protective environment but also improves the efficiency of targeting the drug directly to the cancer cell. It should be pointed out that the chemical formulation of developed hydrogel material for DDS must be properly selected according to the type of cancer, properties of the drug, and their release profile [89,90].

The most commonly used smart polymers in drug delivery purposes are systems sensitive to light, temperature, electric/magnetic field, mechanical stress, ultrasound, and pH [91]. For example, a small change in temperature may cause abrupt modification in the solubility of thermosensitive polymers. The temperature stimulation influences the change of molecular structure from a loose-chain-like to a compacted one, which enables the controlled release of medicinal substances [92]. On the other hand, drug delivery by photosensitive polymers can be realized by one of three major mechanisms, such as photoisomerization or photochemical/photothermal reactions [93].

**Figure 7:**
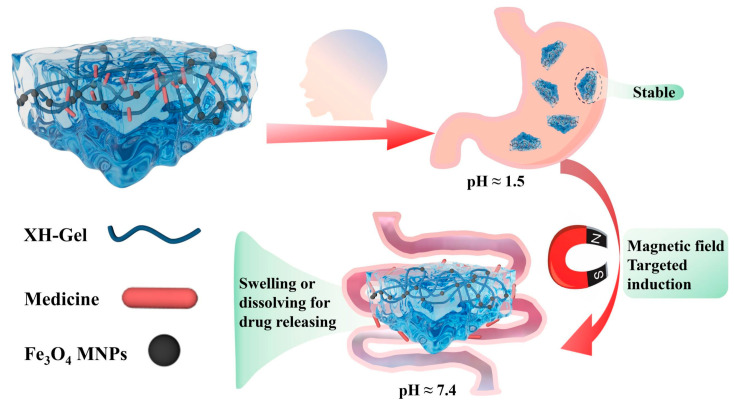
Schematic mechanism of drug control release from pH/magnetic dual-responsive nanocomposite hydrogel in human tissues. Reproduced from Ref. [94], which was published under a CC BY license.

One of the latest achievements in the area of designing smart materials for medicine is nanocomposite hydrogel for drug delivery, described by Long and coworkers [94]. Novel hydrogel synthesized by a combination of xalan hemicellulose with a biodegradable composition based on acrylic acid and poly(ethylene glycol)diacrylate was functionalized with Fe_3_O_4_ magnetic nanoparticles. The obtained polymeric material showed dual-responsivity on pH and magnetic field. The drug release mechanism by the use of novel carbohydrate polymer-based biodegradable pH/magnetic-responsive nanocomposite hydrogel is shown in Figure 7.

Compared to pH-responsive hydrogels, proposed dual-sensitive polymers offer improved capabilities for rapid response and remote control of drug delivery, particularly for gastrointestinal conditions. Additionally, the effect of an external magnetic field extends drug residence time at the target site [94].

**Figure 8:**
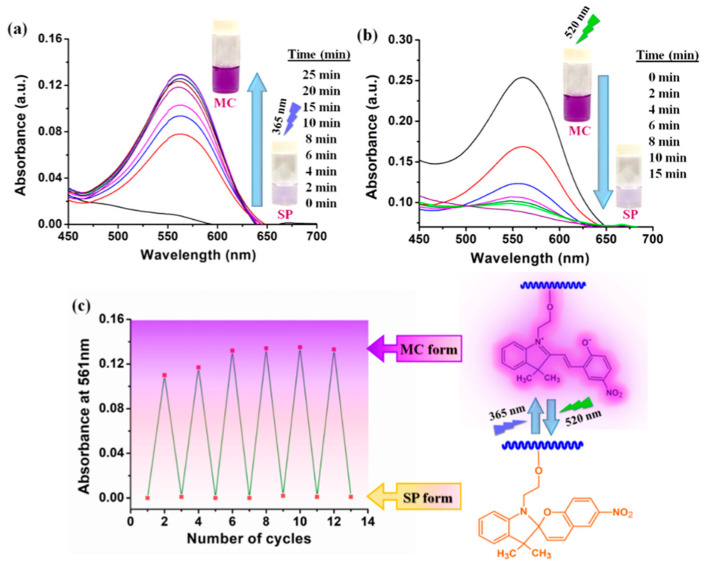
Schematic reaction mechanism of the photoresponsive polymer: (**a**) ring-opening of copolymer (conversion of SP to MC) upon UV light irradiation (365 nm); (**b**) ring-closing (conversion of MC to SP) upon green light irradiation (520 nm) and (**c**) reversibility of reaction of polymer in the result of irradiation of alternate ultraviolet and green light. Reprinted with permission from Ref. [95]. Copyright 2024 American Chemical Society.

Another innovative approach for the application of smart materials in medicine was proposed by Patra and colleagues [95]. The scientists presented a novel photoswitchable smart polymer for gene and anticancer drug delivery for breast cancer treatment. The synthesized copolymer consisted of a hydrophobic core (spiropyran unit, SP, and hydrophilic amino acid moiety as an outer shell. It is common knowledge that some compounds (also spiropyran derivatives) are characterized by high light sensitivity. This feature was taken into account during the design of this smart material. The reaction of polymer on light change was based on the ring-opening and ring-closing mechanism of spiropyran moiety, shown in Figure 8.

The irradiation with UV light leads to the conversion of the colorless nonpolar form of polymer to the hydrophilic merocyanine (MC) form. In other words, 365 nm light induces ring opening in the spiropyran unit, whereas green light (520 nm) leads to ring-closing. It should also be pointed out that the MC form of the novel polymer showed selectivity towards Cu^2+^ ions, which increased concentration is characteristic of breast cancer. For this reason, this smart polymer can be used in the future as a probe for cancer diagnosis and as a nanovehicle for gene and anticancer drug delivery systems in triple-negative breast cancer [95].

Smart polymeric materials play a key role in tissue engineering. This field of regenerative medicine is considered multidisciplinary and interdisciplinary because it combines modern technologies with medical sciences [96]. The major goal of tissue engineering is the development of multifunctional, biocompatible biomaterials for the regeneration of soft (skin, muscles, tendons, skeletal muscles, blood vessels and dental pulp) and hard (bones) tissues. Novel smart polymers can enhance or replace the natural healing processes of tissues, which gives a patient a chance for a full recovery. Biomaterials that are incorporated into the body should be characterized by high biocompatibility, good adhesion, and nontoxicity and should not cause any side effects [96,97,98].

For several years, significant progress in wound treatment using highly effective polymeric materials has been observed. The application of modern products, such as hydrogels, hydrocolloids, foams, and bioadhesives enables the acceleration of the wound healing process [99,100]. A promising alternative to commonly used dressings, stitches and surgical threads is tissue adhesives. These preparations can show sensitivity to external stimuli, such as light, temperature, pH, biomolecules, or electrical field. Moreover, the application of these bioadhesives could minimize the risk of wounds reopening, inflammation, or chronic pain of damaged tissue [101].

**Figure 9:**
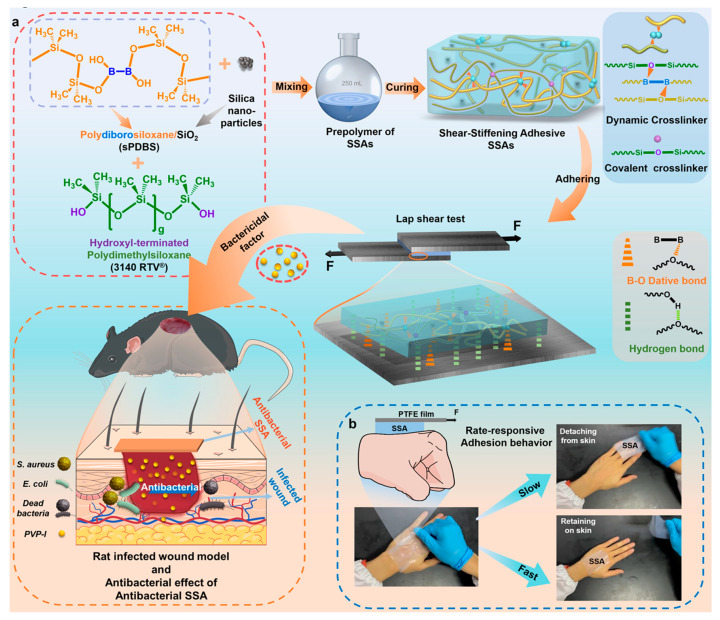
Schematic illustration of (**a**) preparation and application and (**b**) reaction mechanism of external force-responsive bioadhesive. Reprinted with permission from Ref. [102]. Copyright 2024 American Chemical Society.

An example of this type of smart polymer is silicone bioadhesive described by Huang and others [102]. This medical product was synthesized by the combination of shear-stiffening silicon material with commercially available silicone adhesive. The proposed bioadhesive showed sensitivity to external force with on-demand adhesion performance, as shown in Figure 9.

The additional undoubted advantage of this smart polymer was its antibacterial capability, which resulted from the introduction of an appropriate antibacterial factor into the chemical structure of the adhesive [102].

**Figure 10:**
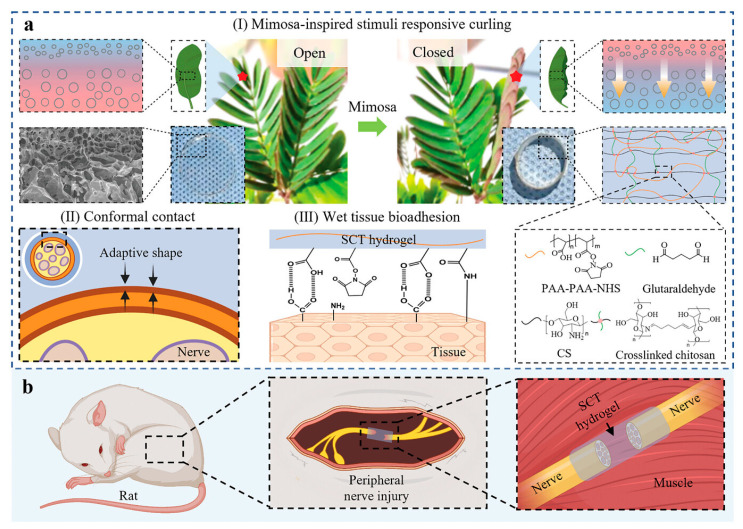
Schematic reaction mechanism of stimuli-responsive (external force, moisture) curling bioadhesive tape: (**a**) major advantages of proposed hydrogel; (**b**) repair process of injured peripheral nerves. Reproduced under terms of the CC-By license [103]. Copyright 2023, Zhang, M.; An, H.; Gu, Z.; Huang, Z.; Zhang, F.; Jiang, B.-G.; Wen, Y., published by John Wiley & Sons, Inc.

Another example of an innovative polymer is hydrogel bioadhesive tape developed by Zhang et al. [103]. This approach can be considered extraordinarily interesting due to the capabilities for repairing possibilities of peripheral nerve injuries by skipping surgical suturing that may cause additional damage. Novel *Mimosa pudica*-inspired smart material was fabricated from chitosan, acrylic acid-*N*-hydroxysuccinimide lipid, and glutaraldehyde. The mechanism of reaction of this polymeric structure is shown in Figure 10.

The proposed smart bioadhesive tape showed changes in its shape, which imitated a mimosa leaf. Thus, it enabled the tight bonding of both sides of the damaged nerve. Moreover, it provided superior flexibility, adaptability, and improved capability to reduce trauma. The rapid absorption of tissue fluid from the nerve surface results in a durable wet-interface adhesion. For this reason, stimuli-responsive polymer has a great potential for clinical applications [103].

**Figure 11:**
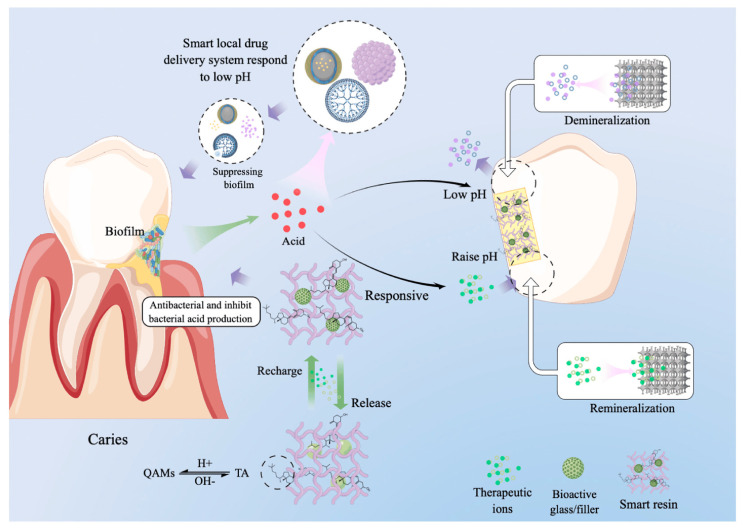
Schematic reaction mechanism of pH-responsive drug delivery system for dental composites. Reproduced with permission from Ref. [106], which was published under a CC BY license.

Smart biopolymers are also used in dentistry. These modern materials can show sensitivity to changes in pH, and presence of bacteria or microorganisms, etc. The application of these materials provides an opportunity to improve standard dental fillings, which are susceptible to many destructive conditions (mechanical stress, bacteria, etc.). New-generation smart polymers include materials that regulate the oral microbial environment, neutralize acids, show antibacterial activity, treat periodontal inflammation, release therapeutic ions, etc. [104,105,106]. The schematic action of smart dental materials is shown in Figure 11.

As shown in Figure 11, bacteria form plaque biofilm tightly adhering to the tooth surface. These bacteria metabolize sugars contained in consumed products, which leads to the production of acids. The acids weaken tooth enamel causing the demineralization of tooth tissues. Smart dental composites respond to pH reduction and release therapeutic ions, which inhibit the growth of bacteria and, thus, lead to long-term remineralization [106,107,108].

New, stimuli-responsive polymers are also designed for ophthalmological purposes. This applies to contact lenses, ocular biomarkers, and various biosensors, which can be used for disease monitoring and therapy [109]. Light-(photochromic) or temperature- (thermochromic) sensitive lenses are well known and have been used in recent decades. Nowadays, scientists and medics are looking for novel multifunctional materials that would indicate sensitivity to other stimuli (intraocular pressure, matrix metalloproteinase-9, bacteria and others) and enable drug delivery [109,110,111,112]. 

An example of the use of smart polymers in ophthalmology is contact lenses proposed by Zhu and colleagues [113]. The developed hydrogel ocular lenses based on a flexible inductor-capacitor-resistor sensor were applied for monitoring intraocular pressure. The research group proved that novel contact lenses do not require the presence of a chip or battery. Moreover, these smart hydrogels showed high sensitivity to pressure changes and have potential applications in medicine [113].

As mentioned earlier, smart polymer materials can be designed to be responsive to changes in the concentration of a specific substance. These types of systems are used as biosensors and play a key role in the diagnostics of various diseases [114,115]. 

**Figure 12:**
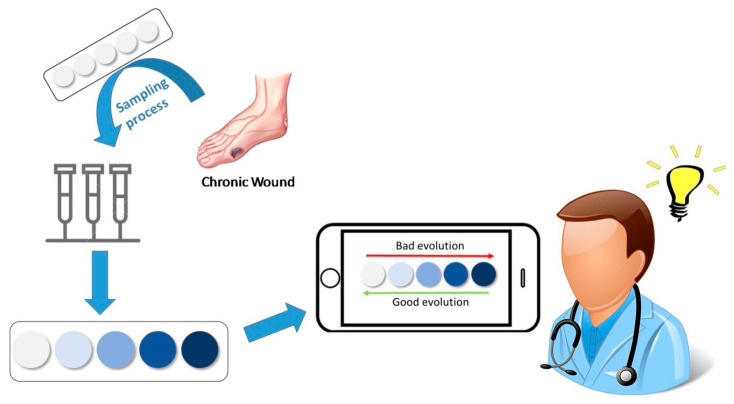
Schematic reaction mechanism of the response of sensor sensitive to the presence of aminoacids in chronic wounds. Reproduced from Ref. [117], published under a CC BY license.

In recent years, biosensors based on polymers have gained importance due to their high sensitivity, stability, precision, and selectivity [116]. For example, Guembe-García and others [117] described a ninhydrin-based sensory polymer and smartphone for monitoring human chronic wounds. The reaction mechanism of a film-shaped polymer in the presence of amino acids is shown in Figure 12.

The principle of operation of the proposed biosensor was quite simple. Upon contact with amino acids, the colorimetric polymeric film changed its color. The analysis of color of the sensory film by the use of a smartphone indicated the dependence of protease activity as the marker for healing disorders [117].

In the literature, the use of smart polymers as biosensors in cancer diagnosis is also described. The stimuli-responsive materials enable quick diagnosis and precise detection of cancer biomarkers, as well as providing better treatment methods [118,119]. In recent years, the potential of using conducting polymers to design biosensors for cancer diagnosis has been noticed and reported [120,121,122].

The abovementioned examples of the application of smart polymers show that this topic is extremely important. Designing novel stimuli-responsive polymers gives a chance for accurate diagnosis and treatment of various diseases. It needs to be highlighted that smart polymers are a new generation of modern materials with wide functionalities and possibilities of application in various medical sectors.

### 6.2. Chemistry

The rapid development of technology and industry is related to an increased demand for smart materials. Polymers that are responsive to various factors can be a promising alternative to popularly used materials, such as glass, ceramics, or metals [123,124,125]. The main areas of application of stimuli-responsive polymers in industry are electronics, mechanics, automotive branches, agriculture, textile production, and others [126,127,128,129,130,131,132,133].

One of the primary goals of engineering sciences is the development of new materials, which are necessary for the production of advanced devices. Smart polymeric materials are used in the manufacturing of various types of sensors, actuators, conductors, etc. [4,134,135,136]. The most typical stimulus-sensitive materials are polymers that respond to changes in external force, electric field, and concentration of specific substances [137].

The massive industrialization, modernization, and agricultural activity can contribute to an increased release of toxic substances into the environment. For this reason, developing highly effective sensing devices, as well as novel purification technologies, is very important for ensuring the safety of human health and the environment. The use of sensors for detecting harmful substances, i.e., gases (CO, CO_2_, NO_2_, NH_3_, H_2_S, SO_2_, etc.), heavy metal ions (Hg^2+^, Pb^2+^ and others), pesticides, and various organic compounds enable controls of their concentration in the environment [138,139]. It is expected that designed monitoring devices will show high sensitivity, selectivity, and efficiency. Significantly, the application of smart polymers in the production of various types of sensors can enhance their effectiveness of working [140].

**Figure 13:**
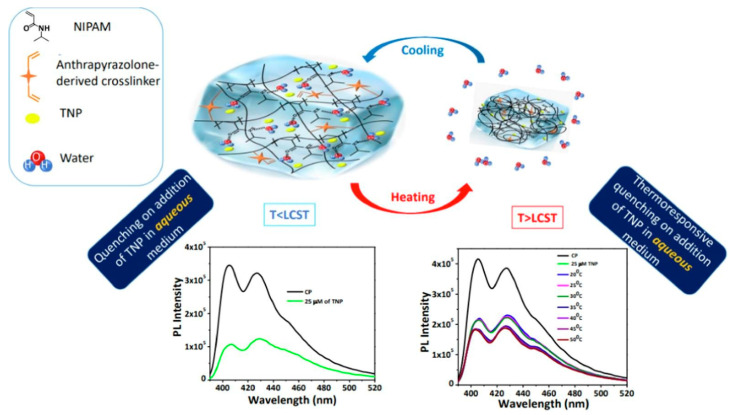
Schematic reaction mechanism of hydrogel sensor sensitive to the presence of nitroaromatic compounds. Reproduced from Ref. [142] with permission from Elsevier, Copyright 2023.

In recent years, many articles describing novel polymeric sensors and detectors or actuators have been published [141]. For example, Babu et al. [142] proposed a smart polymeric sensor for the detection of nitroaromatics in an aqueous medium. This polymeric structure based on poly(*N*-isopropylacrylamide) and anthrapyrazolone showed sensitivity to the presence of various compounds that are components of explosives, i.e., *p*-nitrophenol, 2,4-dinitrophenol, 2,4,6-trinitrophenol, etc. Significantly, the novel sensor also reacted to changes in temperature, which is shown schematically in Figure 13.

Generally, the presence of nitroaromatic compounds in the water resulted in a reduction of emission intensity of the polymeric probe, which was attributed to a photoinduced electron transfer process occurring between the thermoresponsive crosslinked polymer and the detected compound. Moreover, the additional fluorescence quenching effect was observed in higher temperatures beyond the lowest critical solution temperature, which resulted from the insolubility of poly(*N*-isopropylacrylamide) in water [142].

The latest achievements in the design of stimulus-responsive materials include polymeric sensors for fluoride ions and Alizarin Red S dye proposed by Zheng and colleagues [143]. The research group designed a stimuli-responsive luminescent polymer containing borinic acid moiety. The developed detector showed great application potential due to the possibility of also monitoring 8-hydroxyquinoline, which is commonly used in the production of various pharmaceutical agents.

Polymers showing sensitivity to pH can also be used for the selective detection of trace amounts of precious metal ions from different sources [144]. For example, Yang and co- [145] proposed porous graphene-like carbon hydrogel for highly effective recovery of Ag ions from aqueous media. The efficiency of Ag^+^ adsorption increased gradually with acidity decreasing and achieved maximum at pH = 6. On the other hand, Sharma et al. [146] developed fluorescent imidazolium hydrogels for recovering platinum from spent auto catalysts. Importantly, the proposed smart polymer showed high effectiveness across a wide pH range. Moreover, the recovered platinum was of high purity (about 96%).

Smart polymers are suitable for removing various pollutants from industry and households. Most often, these materials play the role of membranes retaining harmful substances [147,148]. Many review articles summarize progress in the field of stimuli-responsive polymers used for environmental safety and protection. For example, Musarurwa and Tavengwa [149] presented a review paper on recyclable polysaccharide/stimuli-sensitive polymeric composites. These polymers can be used for water remediation processes. On the other hand, Zhang and others [150] developed a smart coating for the separation of water and oil. The described polymeric materials showed dual responsivity to both photon and pH stimuli. The incorporation of photosensitive segments into a polymer structure contributed to the sensitivity of light. This new generation material enabled separation in mild conditions, easy process control, and is characterized by the absence of secondary pollution.

**Figure 14:**
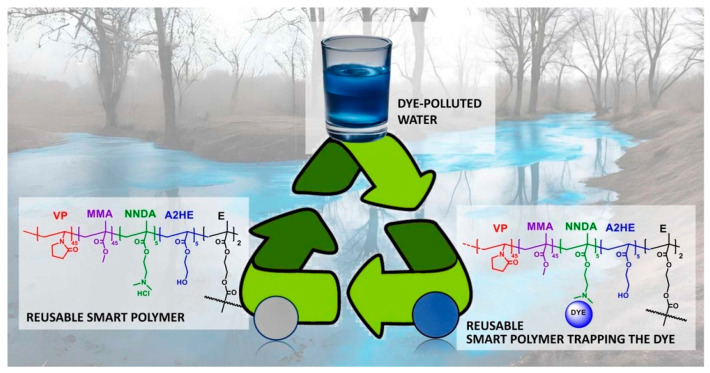
Schematic reaction mechanism of acrylic film sensitive to the presence of textile industry pollutants. Reproduced from Ref. [151] with permission from Elsevier, Copyright 2024.

Similarly, an interesting approach to removing pollutants from the environment was described by Guembe-García et al. [151]. The research group presented reusable acrylic film for the efficient extraction of anionic dyes. As illustrated in Figure 14, the polymeric material shows specific interactions with a wide group of textile dyes. Importantly, a removal percentage efficiency above 90% was obtained. Moreover, this smart membrane could be used at least five times and, compared to the currently used separation materials, was characterized by better thermal and mechanical properties, enhanced manageability, and durability.

**Figure 15:**
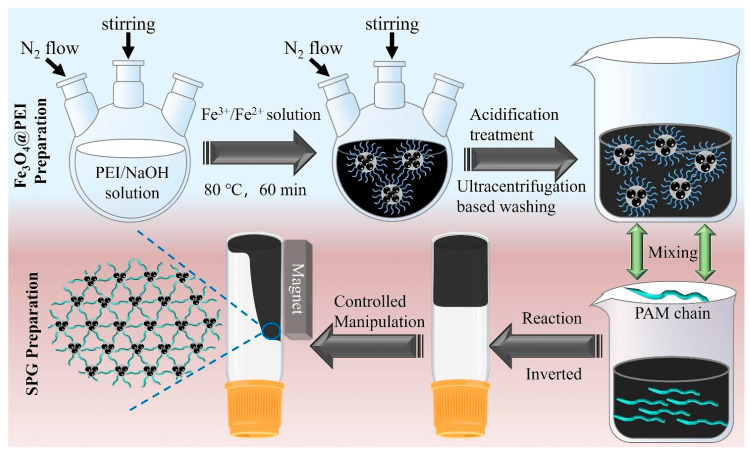
Schematic reaction mechanism of magnetic field-responsive polymeric gel. Reproduced from Ref. [152] with permission from Elsevier, Copyright 2024.

Another example of functional stimuli-responsive polymers is the magnetic smart polymer gel proposed by Wang and coworkers [152]. The gel technology is recognized as one of the most important and highly effective methods for enhanced oil recovery. For this reason, this advantage was taken into account during the design of a novel gel system. The modern polymeric material was composed of polyacrylamide and poly(ethylenimine) functionalized by Fe_3_O_4_ nanoparticles. The mechanism of action of this smart polymer is shown schematically in Figure 15.

The polymeric gel system showed good viscoelastic properties, stability in aqueous media, controllable efficiency, and high-temperature resistance. Moreover, its sensitivity to magnetic field changes enabled easy and repeatedly moving to a specific location. Therefore, the proposed smart polymer can be useful for directional plugging in oil fields.

**Figure 16:**
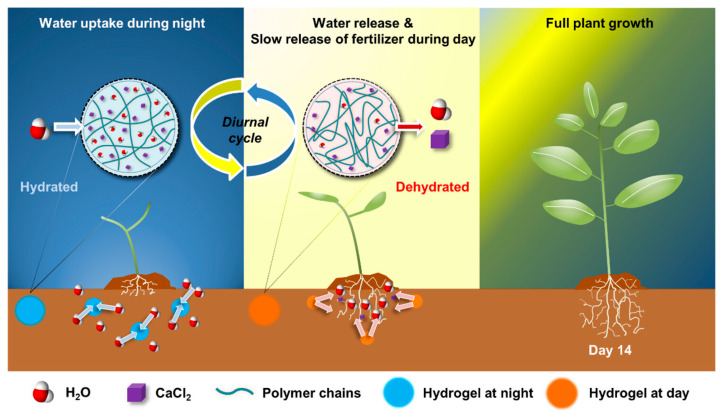
Schematic reaction mechanism of moisture-sensitive hydrogel in a diurnal cycle. Reprinted with permission from Ref. [156]. Copyright 2024 American Chemical Society.

As mentioned earlier, stimuli-responsive materials are also useful in agriculture. The polymeric systems can play a role in the controlled delivery systems of pesticides, herbicides, or fertilizers, as well as super-absorbents, soil conditioners, and hydrogels [153,154,155]. In order to search for more efficient water and nutrient utilization, according to the principles of sustainable agriculture, Park and others [156] developed self-irrigation and slow-release fertilizer hydrogels. As depicted in Figure 16, the smart polymer demonstrated diurnal functionality. At night, hydrogel absorbed water vapor. Then, as a result of the phased transition of the polymer matrix, the accumulated water during the day and CaCl_2_ were released. This action provided gradual soil irrigation and thus, effectively released nutrients.

The discussion of using smart polymeric materials in various areas should take into account the chemical industry, including the production of various types of coatings. The polymeric coatings applied to miscellaneous materials, such as wood, metals, etc. can play not only a decorative role but also protection for various external factors (light, microorganisms, chemicals, water, oxygen and many others). Additionally, they can show responsivity towards different stimuli and thus offer better functionality [157,158,159,160,161]. 

Polymeric formulations that can be successfully classified into smart materials are pressure-sensitive adhesives (PSAs) or films. The PSA is a type of adhesive that forms a bond with the surface when an external force (pressure) is applied. Apart from its ability to combine various materials, PSAs can show additional functional features. These polymers might be responsive to temperature, pH, light, ionic strength, magnetic/electric field, etc. [162,163].

**Figure 17:**
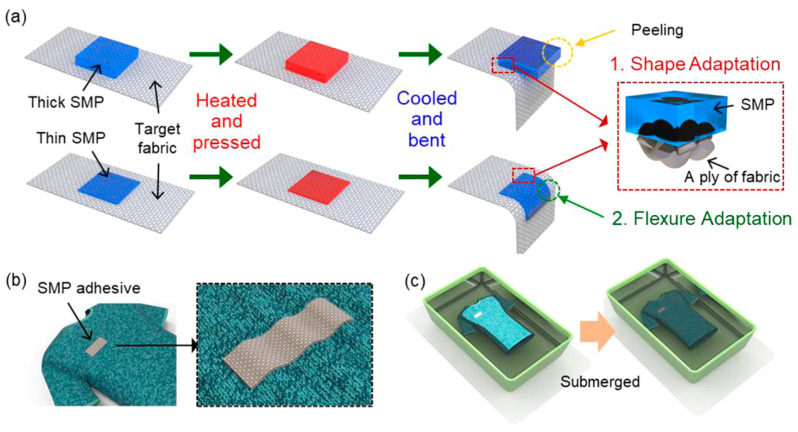
Schematic reaction mechanism of shape and flexure-sensitive shape-memory polymer adhesive: (**a**) effect of adhesive thickness on the shape-memory ability; (**b**) adaptation of smart polymer to fabric; (**c**) waterproof and underwater properties of SMP adhesive. Reprinted with permission from Ref. [166]. Copyright 2021 American Chemical Society.

An example of a smart polymer is thermally conductive PSA presented by Cui and others [164]. The proposed adhesive showed excellent adhesion properties and UV-sensitive peelability. Ren and colleagues [165] synthesized humidity-insensitive waterborne polyurethane PSA based on biobased castor oil and 3-aminopropyl triethoxysilane. On the other hand, Son and Kim [166] designed a shape memory polymer adhesive that was able to adhere to various flexible surfaces like fabrics. The action mechanism of this smart polymer is shown in Figure 17. The proposed material was characterized by strong adhesion, shape, and flexure adaptation in both dry and underwater conditions.

The conception of designing polymeric materials with the ability to self-heal can be considered a huge breakthrough in the area of smart materials [167,168]. Drawing inspiration from nature (e.g., regeneration of bone or skin injuries), new polymers that can repair damage have been developed. Polymeric materials can activate an external stimulus (light, pressure, temperature, magnetic/electric field, etc.), which leads to a self-healing reaction [169].

**Figure 18:**
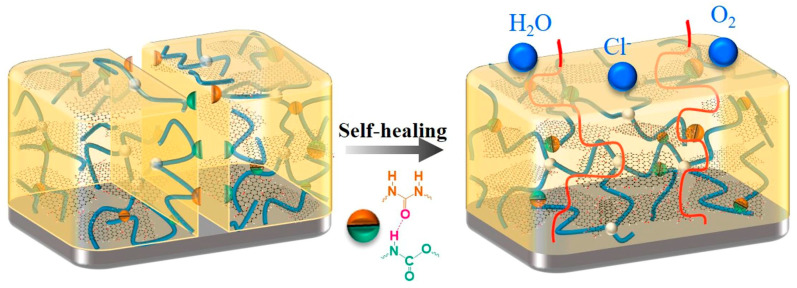
Schematic self-healing mechanism of a corrosion-resistant polyurethane coating. Reproduced from Ref. [174] with permission from Elsevier, Copyright 2024.

There are many examples of smart stimuli-sensitive coatings described in the literature [170,171,172,173]. One of the recent developments in the area of smart polymers is polyurethane coating, proposed by Pang and coworkers [174]. The enhancement of polyurethane by graphene oxide increased the corrosion resistance of the coating. The self-healing ability was attributed to the reversible hydrogen-bonding interactions between urethane groups and urea units (Figure 18). Moreover, the introduction of glycerol moieties into the polymer structure improved the mechanical properties of the polymer coating.

Another example of stimuli-sensitive polymer coatings is acrylate formulations described by Paquet and others [175]. The UV-curable polymerizable mixtures consisted of acrylate monomers and acrylate oligomers containing hydroxyl groups. The proposed coatings showed high potential for use in wood surface protection. The research group proved that the highest degree of self-healing of coatings can be observed for formulations containing components characterized by low steric hindrance and have a high number of hydrogen bonds. The self-healing process was induced by physical stimulus—increased temperature (80 °C, heating time: 2 h).

One of the latest reports on the topic of smart polymers is epoxy coatings developed by Wu and colleagues [176]. Novel oligomers containing disulfide bonds were synthesized in the reaction between bisphenol A glycidyl ether and 3,3′-dithiodipropionic acid. The addition of dimeric acid gave a series of reprocessable epoxy resins showing self-healing ability, excellent mechanical properties, and corrosion resistance. The highest degree of self-healing was achieved at about 94% (within 1 h at 60 °C).

To summarize, designing polymeric materials for industry is extremely important. Various smart polymers can be a good alternative for commonly used materials. Moreover, due to stimuli-responsivity, these polymers provide greater opportunities for application in various technologies.

### 6.3. Modern Technologies

Nowadays, worldwide attention is focused on the search for new, functional materials, which can be used in the production of high-tech systems and devices. The rapid progress of modern technologies and artificial intelligence (AI) affects the increasing demand for smart polymeric materials [177,178].

**Figure 19:**
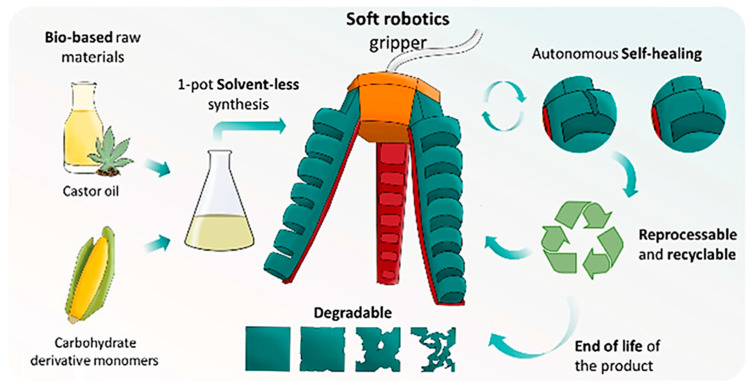
Schematic illustration of preparation and autonomic self-healing reaction of elastomer. Reprinted with permission from Ref. [183]. Copyright 2023 American Chemical Society.

In recent years, a remarkable interest in soft robotics has been observed. The scientists are seeking to carry out a veritable technological breakthrough in this area [179,180,181,182]. For example, Cornellà et al. [183] presented elastomers for sustainable robotics. The proposed polymer material showed a number of beneficial features, such as autonomous self-healing ability, and being recyclable and degradable. The polymeric matric was composed of biobased raw materials and carbohydrate derivative monomers. The newly developed polymer was used for the production of a pneumatic gripper (Figure 19) for soft robotics applications.

**Figure 20:**
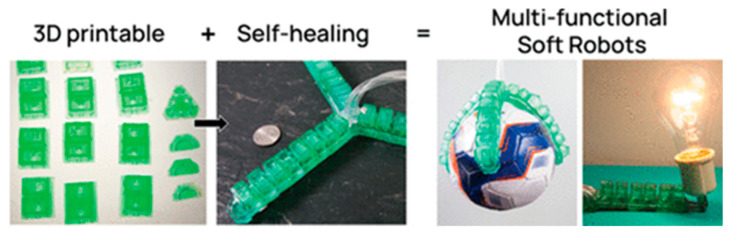
Schematic illustration of developed self-healing elastomers. Reprinted with permission from Ref. [184]. Copyright 2021 American Chemical Society.

An equally interesting invention was proposed by Gomez and colleagues [184]. The scientists described using of self-healing elastomers in 3D printing technology. The used photosensitive elastomer resins exhibited ultra stretchability and repeatable self-healing capacity. The printed polymer showed high potential for application in soft robotics (Figure 20).

**Figure 21:**
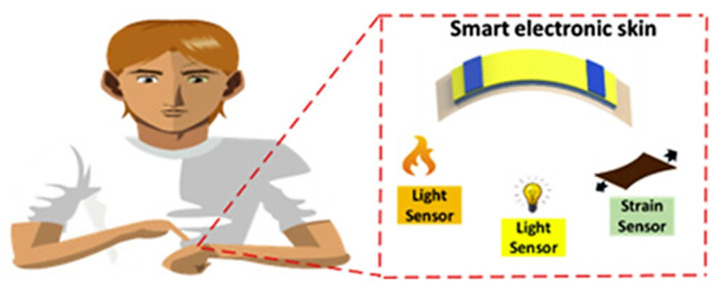
Schematic illustration of light- and strain-responsive polymeric film for smart electronics. Reproduced from Ref. [189] with permission from Elsevier, Copyright 2020.

The novelty in the field of smart polymers is electronic skin (e-skin) [185,186]. Currently, this advanced material is a matter of intensive investigation because of a wide range of applications in soft robotics, virtual reality, biointegrated electronics, intelligent gloves, and many others [187]. The e-skin is a highly integrated and advanced electronic system that can convert various types of external stimuli (i.e., pressure, humidity, deformation, chemical substances, etc.) into electric signals. For this reason, this material attracts the attention of many researchers [188]. An example of this type of smart polymer could be multifunctional electronic skin developed by Ahmed and others [189]. This polymeric film was a self-powering material, which showed energy conversion capability. As illustrated in Figure 21, this device was capable of the detection of light and showed strain-sensitivity.

Advanced polymeric materials also include smart hybrid textiles. A suitable integration of smart polymers into textiles has led to obtaining novel functionalities for these materials [190]. Smart polymeric textiles can be used for monitoring body movements and the degree of sweat secretion, as well as showing ultraviolet/radioactive irradiation/temperature/antibacterial/antivirus, etc. resistance and many others [191,192].

**Figure 22:**
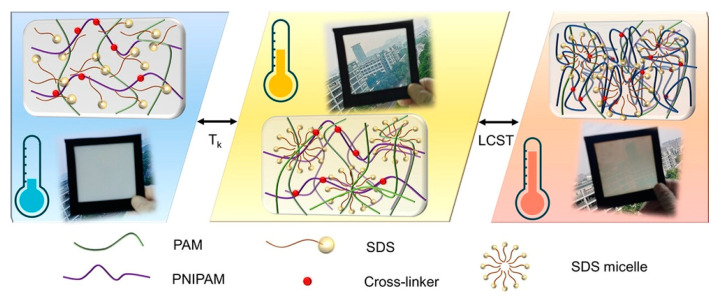
Schematic reaction mechanism of dual (light- and temperature-) responsive hydrogel. Reprinted with permission from Ref. [195]. Copyright 2022 American Chemical Society.

The increased efforts in the area of sustainable energy management research have led to the development of advanced, energy-saving devices. Although dye-sensitized solar cells (DSSCs) have been well-known for many years, new solutions, which would enable their improved efficiency have been designed [193,194]. An interesting approach for modern energetic technologies is thermochromic smart windows. This type of stimuli-sensitive polymeric material was proposed by Dai and others [195]. As shown in Figure 22, the basis of the described innovation was a new dual-responsive hydrogel, which exhibited a satisfactory solar modulation ability. The prepared hydrogel was composed of poly(N-isopropylacrylamide)—PNIPAM, polyacrylamide—PAM and contained sodium dodecyl sulfate (SDS). In comparison with traditional windows, smart polymeric material offers better thermal insulation and heat preservation. The control of temperature from low to medium and high, this hydrogel showed a three-stage transition of opaque-transparent-translucent. This action mechanism makes it an ideal candidate for smart windows.

A similar advanced hydrogel system for smart windows was described by Li and coworkers [196]. The scientists also took into account the sensitivity of polymers to temperature changes. Moreover, this material is based on thermosensitive shape memory polymer and an optical film and was able to reversibly transform shape like a butterfly wing.

The described examples of novel smart polymeric materials show only a small part of the new achievements in this field. The technology development progress, as well as the multidisciplinary approach, gives a huge chance for another revolution in the area of a new generation of modern stimuli-responsive materials.

## 7. Future Perspectives

Although smart polymers have been known for many years, their potential has only recently been well understood. The stimuli-responsive materials are widely used in various fields. For this reason, searching for novel advanced systems is necessary for the further development of high technologies [197,198].

Depending on the area of application of smart polymers, these materials must fulfill various requirements. For example, stimuli-sensitive polymers used in the medical field should be biocompatible, nontoxic, not mutagenic, and highly sensitive to various factors. Moreover, the design and development of novel smart polymers will be important for the effective treatment of various diseases, such as cancer. On the other hand, smart materials dedicated to soft robotics and the automotive industry should be highly durable and have high resistance to harmful external factors, etc. [199,200].

The presented literature review confirms the great interest in the topic of smart polymers. Future work should focus on further understanding the mechanisms of polymer reaction to stimuli. The aim is to create materials that can recognize and respond to many types of stimuli simultaneously and adapt this response depending on the presence of these signals. Certainly, the development of artificial intelligence will set new trends for the future generation of smart materials.

## 8. Conclusions

The changing needs of society require the creation of new products that are useful in everyday life. The answer in polymer chemistry is smart materials. The development of this field of polymers allows for better adaptation to the needs. The demonstrated use of these materials confirms that their development can revolutionize many industries. The interest of groups of scientists in the development of this field has already been visible for many years.

## Figures and Tables

**Table 1:** Advantages and disadvantages of using smart polymers [8].

Advantages	Disadvantages
Biocompatible, robust, flexible, easy to color, mild—cause fewer complications for patients.	There are difficulties in sterilizing them.
Facilitate dosing for patients—possibility of producing individualized products, e.g., tablets.	Lack of high mechanical resistance.
Simple synthesis method.	Sometimes it is difficult to load drugs and cells in a ready-made matrix.
They support/facilitate the transport of ingredients into cells.	
Provide prolonged drug release time and cause fewer side effects.	
